# Folinic acid in colorectal cancer: esquire or fellow knight? Real-world results from a mono institutional, retrospective study

**DOI:** 10.18632/oncotarget.27872

**Published:** 2021-02-02

**Authors:** Francesco Jacopo Romano, Carmela Barbato, Maria Biglietto, Vincenzo Di Lauro, Dario Arundine, Roberto Fiorentino, Francesca Ambrosio, Maresa Cammarota, Bruno Chiurazzi, Livio Puglia, Sarah Scagliarini, Raffaella Ruocco, Carmen Mocerino, Ivana Cerillo, Maria Fiorella Brangi, Ferdinando Riccardi

**Affiliations:** ^1^Antonio Cardarelli Hospital, Oncology Unit, Naples, Italy; ^2^Federico II University, Department of Medicine and Surgery, Oncology Unit, Naples, Italy; ^3^Antonio Cardarelli Hospital, Pharmacy Unit, Naples, Italy

**Keywords:** colorectal cancer, folinic acid, sodium levofolinate, thymidylate synthase, non oncogene addiction

## Abstract

The stock of therapeutic weapons available in metastatic colorectal cancer (mCRC) has been progressively grown over the years, with improving both survival and patients' clinical outcome: notwithstanding advances in the knowledge of mCRC biology, as well as advances in treatment, fluoropyrimidine antimetabolite drugs have been for 30 years the mainstay of chemotherapy protocols for this malignancy. 5-Fluorouracil (5FU) seems to act differently depending on administration method: elastomer-mediated continuous infusion better inhibits Thymidylate Synthase (TS), an enzyme playing a pivotal role in DNA synthetic pathway. TS overexpression is an acknowledged poor prognosis predicting factor. The simultaneous combination of 5FU and folinate salt synergistically strengthens fluorouracil cytotoxic effect. In our experience, levofolinate and 5FU together in continuous infusion prolong progression free survival of patients suffering from mCRC, moreover decreasing death risk and showing a clear clinical benefit for patients, irrespective of RAS mutational status, primitive tumor side and metastases surgery.

## INTRODUCTION

Notwithstanding advances in the knowledge of metastatic colorectal cancer (mCRC) biology, as well as advances in treatment, fluoropyrimidine antimetabolite drugs (5-fluorouracil – FUra, 5FU, 5-fluoro-2’-deoxyuridine – 5FUdR, 5’-deoxy-5-fluoro-N-[(pentyloxy)carbonyl]-cytidine – capecitabine, an oral FUra prodrug) are currently the mainstay of chemotherapy protocols for this malignancy. 5FU seems to act differently depending on administration method: quick bolus mainly increases incorporation of 5FU in RNA [1], even yielding a more severe hematological and gastrointestinal toxicity than continuous infusion [2], whereas elastomer-mediated continuous infusion long inhibits Thymidylate Synthase (TS) [3, 4]. TS is an enzyme playing a pivotal role in DNA synthetic pathway by producing a crucial nucleotide for genome integrity and cellular economy: Colon Rectal Cancer (CRC) cells exhibit a large *non-oncogene addiction* toward TS, and its overexpression in CRC cells is an acknowledged poor prognosis predicting factor [5, 6].

**Figure 1:**
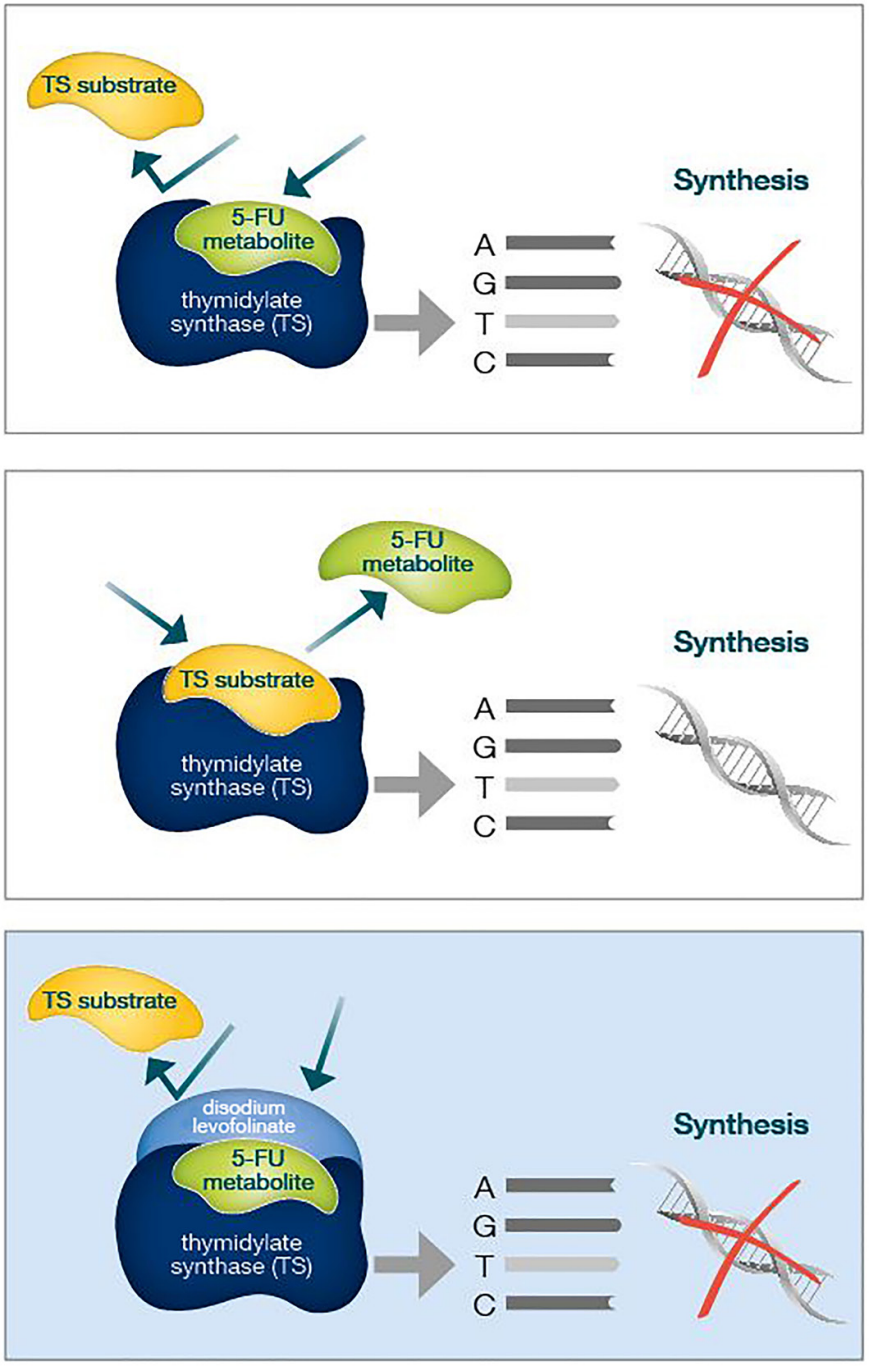
Folinic acid acts as a "molecular cap", strengthening the binding of 5FU to TS and therefore better inhibiting its enzymatic activity. TS overexpression in CRC cells is an acknowledged poor prognosis predicting factor.

Preclinical evidence has pointed out that culture medium containing folinic acid increases 5FU-mediated cell growth inhibition and cytotoxicity (Figure 1). Actually, folinic acid acts as a “stabilizer” of fluoropyrimidines and TS, with accumulating inactive complexes [7, 8]. Modulation of 5FU activity has been studied for several years, with the aim to enhance antineoplastic effect by combining bolus and continuous infusion administration to maximize 5FU antitumor efficacy. However, 5FU incorporation into RNA and DNA of tumor tissue seems to not correlate with treatment efficacy, unlike TS inhibition [9].

Therefore, therapeutic strategies combining bolus and continuous infusion have been made to better exploit both genotoxic effect of fluoropyrimidines incorporation and TS inhibition, with extending infusion time to 48 hours and adding folinic acid [10, 11].

Levofolinate (LF) is the active stereoisomer of folinic acid (FA), and its calcium salt (CaLF) is widely used in HD-FUFA regimen, the so-called “*De Gramont regimen*”, which currently represents the backbone of metastatic Colon Rectal Cancer (mCRC) therapy, alternatively adding oxalilplatin, irinotecan or biological agents, such as anti-EGFR or antiangiogenic monoclonal antibodies. Usually, 5FU bolus is administered at the middle of a 2-hours folinic acid infusion. Unfortunately, calcium-salt levofolinate quickly precipitates when co-administered with 5FU in continuous infusion pump, with catheter clogging and limiting its use to bolus-concomitant administration [12, 13].

As easily deduced from preclinical studies highlighted until now, prolonging simultaneous exposure of cancer cells to 5FU and folinic acid could imply a greater TS inhibition and thus a greater cell death extent. Indeed, both in cell cultures and in “vivo” animal models, only the simultaneous combination of 5FU and folinate salt synergistically strengthens fluorouracil cytotoxic effect [14]. Disodium LevoFolinate (NaLF) is a compound with similar pharmacological features of CaLF, but more soluble. For this reason, NaLF can be safely administered mixed with 5FU in a single pump without the risk of precipitation and catheter occlusion [15]. In addition we can reach an even shorter administration time for the two drugs, requiring fewer human resources compared with sequential administration, less discomfort for patients and more compliance to treatment.

To date, no study has been made comparing the sequential standard treated with CaLF to the concomitant 5FU-NaLF regimen. Our retrospective, single-center observational study is the first with the aim of evaluating differences between these administration modalities: in particular, we wondered if co-administration of 5FU and folinic acid in continuous infusion was as effective as the classic sequential administration, or even more effective in terms of progression free- and overall survival, especially considering the aforementioned preclinical data.

## RESULTS

The number of patients treated with therapy based on calcium-levolinate and sodium-levolinate were 105 and 95, respectively: between the two groups, no statistically significant difference has been found, but median follow up duration – 28,8 for CaLF vs 18,8 months for NaLF, *p value 0,0001* (Mann-Whitney *U* Test). This finding should not be surprising, because of the more recent introduction of sodium levofolinate in clinical practice. Noteworthy, in the NaLF group there was a greater frequency of RAS mutated-patients than in CaLF counterpart, although not statistically meaningful – 62% vs 50%, *p value* 0,074 (Table 1).

**Table 1:** Between the two study groups, no statistically significant difference has been found, but median follow up duration - 28,8 for CaLF vs 18,8 months for NaLF, *p* value 0,0001 (Mann-Whitney *U* Test)

	Sodium 95 pts		Calcium 105 pts		*p* value
	N°	%	N°	%	
Male	60	63	60	57	0.386^*^
Female	35	37	45	43	
Age at first line (median)	65,6 yrs		65,1 yrs		0.878^**^
Left colon cancer	63	66	66	63	0.610^*^
Right colon cancer	32	34	39	37	
ALL RAS Wild Type	36	38	53	50	0.074^*^
KRAS/NRAS Mutated	59	62	52	50	
Metastases Surgery					
No	72	76	83	79	0.699^*^
Yes	23	24	23	21	
Chemotherapy scheme					
HD-FUFA	7	8	2	2	0.094^*^
FOLFOX	63	66	81	77	
FOLFIRI	25	26	22	21	
No biological agent	3	3	7	7	0.066^*^
Anti EGFR Mab	29	31	45	43	
Anti VEGF Mab	63	66	53	50	
Follow up duration (median)	**18, 8 months**		**28, 8 months**		0.0001^**^

^**^Mann-Whitney *U* Test, ^*^Pearson χ^2^ Test. This finding should not be surprising, because of the more recent introduction of sodium levofolinate in clinical practice. Noteworthy, in the NaLF group there was a greater frequency of RAS mutated-patients than in CaLF counterpart, although not statistically meaningful - 62% vs 50%, *p* value 0,074.

**Figure 2:**
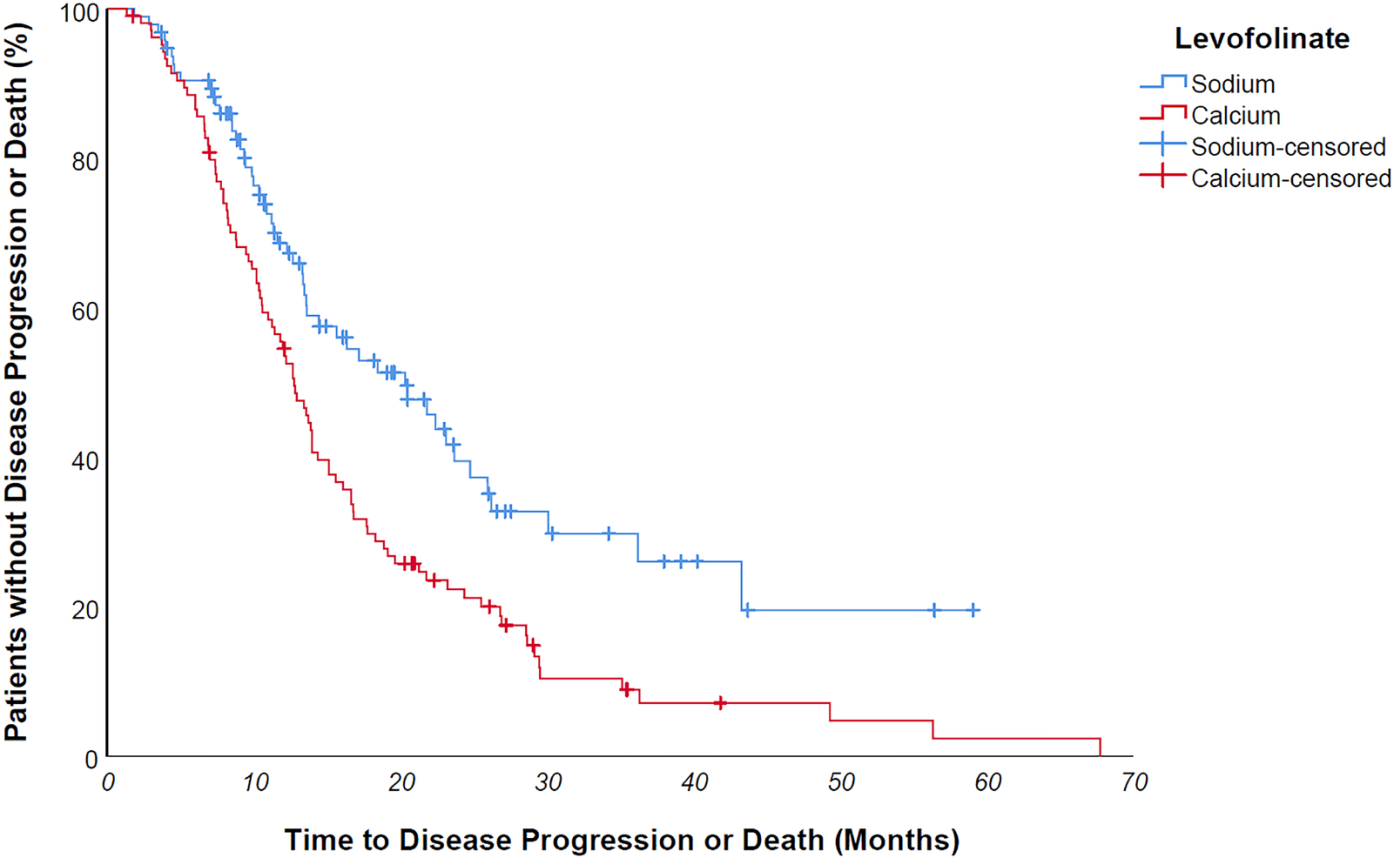
Median progression free survival of patients undergoing sodium levofolinate-based therapy was significantly longer than patients treated with calcium folinate - 20,3 versus 12,8 months, *p* value 0,001.

**Figure 3:**
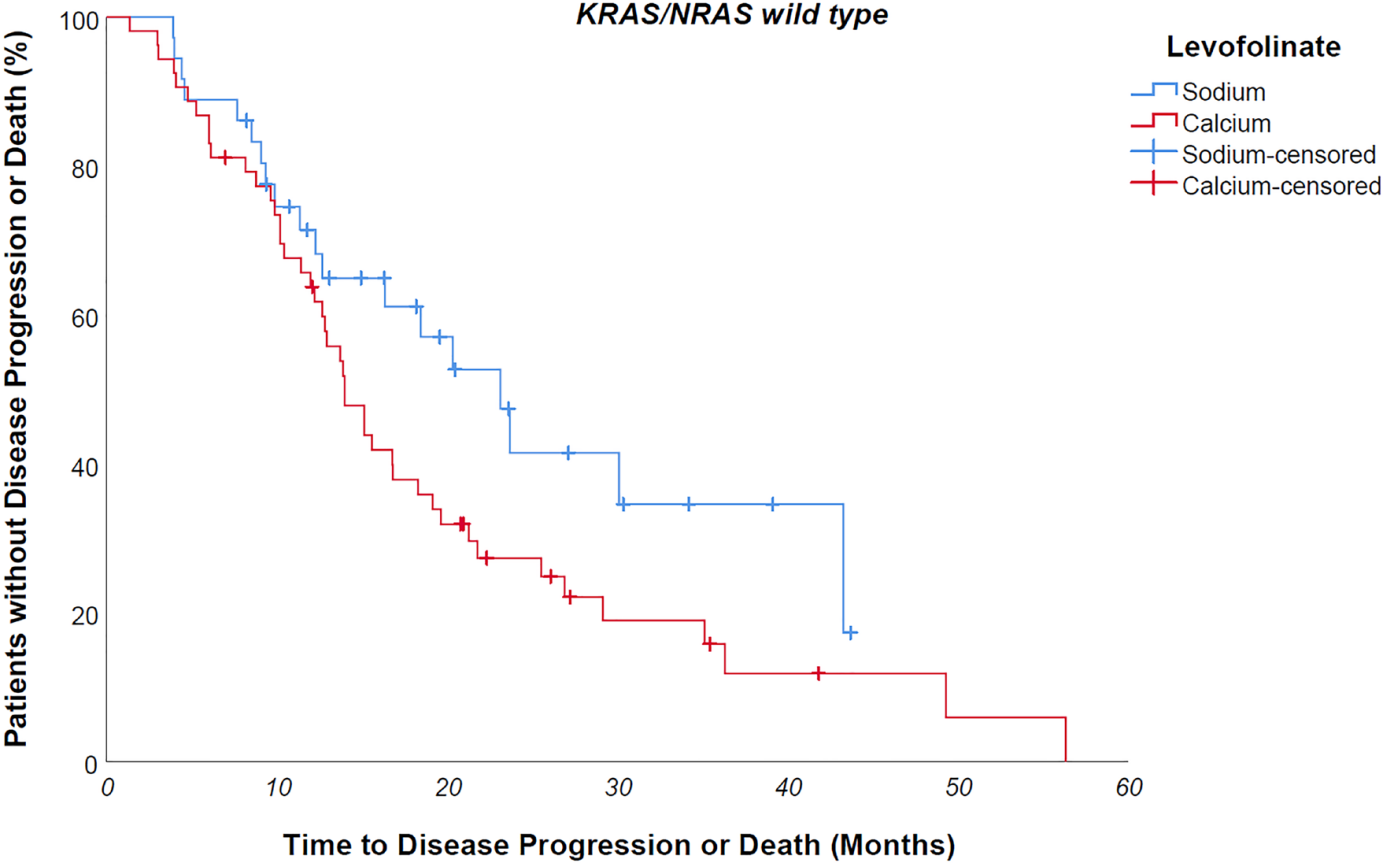
KRAS/NRAS wild type NaLF treated patients progressed 9 months later than CaLF treated counterpart, though not in a statistically significant way (median PFS 23,1 vs 14 months, *p* value 0,085).

**Figure 4:**
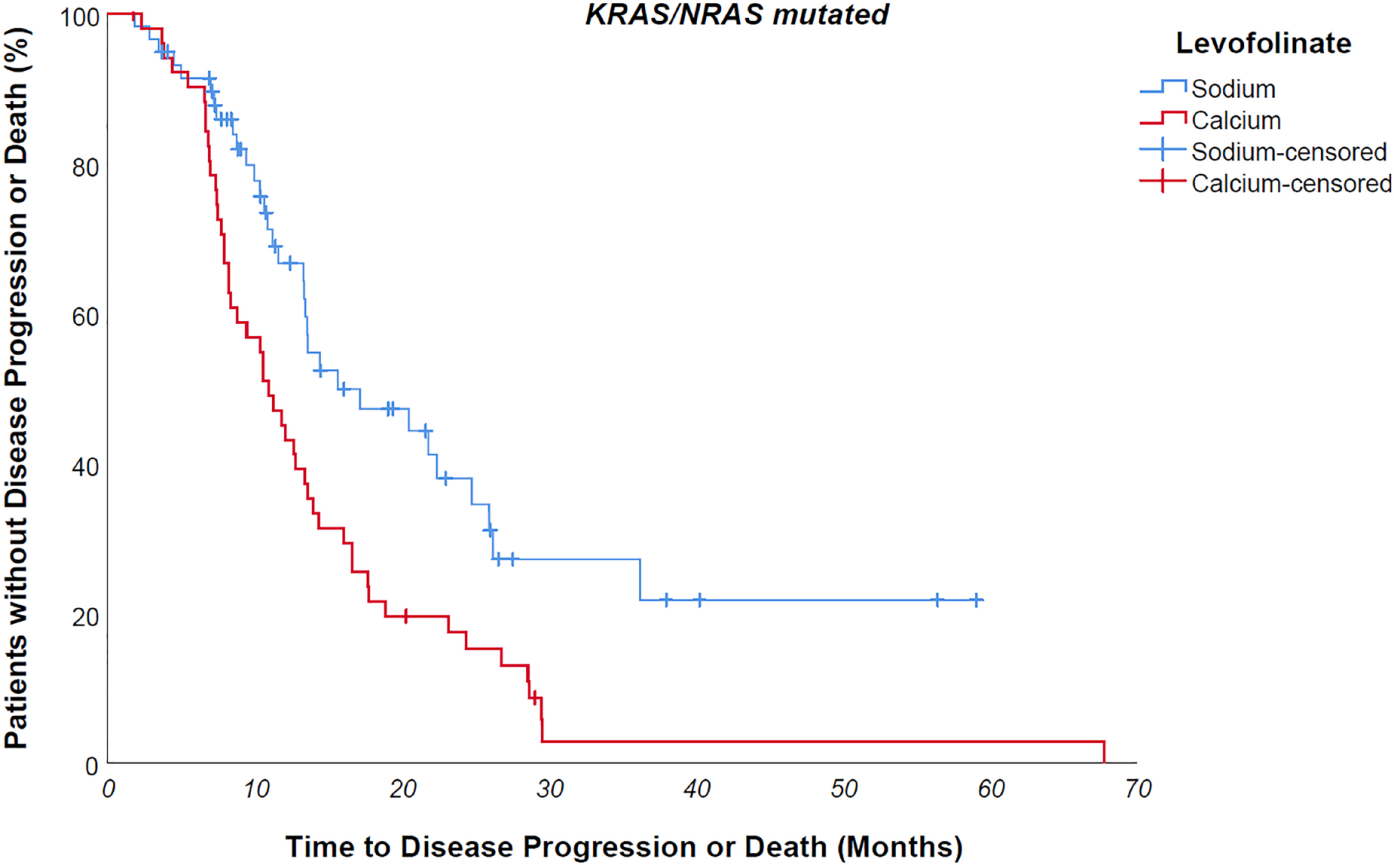
KRAS/NRAS mutated NaLF treated patients progressed 4.7 months later than CaLF treated patients (median PFS 15.7 vs 11 months, *p* value 0,003).

Median progression free survival of the entire study population was 14 months. Median progression free survival of patients undergoing sodium levofolinate-based therapy was significantly longer than patients treated with calcium folinate – 20,3 versus 12,8 months, *p value 0,001* (Figure 2) As easily predictable, patients suffering from a *KRAS/NRAS mutated* mCRC progressed earlier than wild-type counterpart, although without a clear statistically meaningful difference – 13,4 vs 16,3 months, *p value 0,183*. KRAS/NRAS wild type NaLF treated patients progressed 9 months later than CaLF treated counterpart, though not in a statistically significant way (median PFS 23,1 vs 14 months, *p value 0,085*); conversely, *KRAS/NRAS mutated* NaLF treated patients progressed 4.7 months later than CaLF treated patients (median PFS 15.7 vs 11 months, *p value 0,003*) (Figures 3 and 4).

In Cox multivariate analysis for PFS, NaLF halved the risk of progression – HR 0,5 - *p value 0,0002*: the only other significant factor in Cox multivariate was chemotherapy scheme: both FOLFIRI and FOLFOX decreased progression risk compared to HD-FUFA only therapy (Table 2).

**Table 2:** PFS Cox univariate and multivariate

Cox model	Univariate			Multivariate		
	HR	CI 95%	*p* value	HR	CI 95%	*p* value
Gender (M vs F)	1.016	0.724–1,426	0.927	1.042	0,730–1,486	0.82
Age at first line(continuous)	1.002	0.985–1.019	0.809	0.995	0,978–1,013	0.59
Tumor side (left colon vs right colon)	0,733	0.517–1.04	0.082	0.774	0,521–1,148	0.2
ALL RAS mutational status (WT vs mutated)	0.799	0.573–1.113	0.184	0.959	0,470–1,957	0.9
Metastases Surgery (Yes vs No)	0.940	0.630–1.403	0.762	0.978	0,639–1,498	0.92
**Chemotherapy scheme**			**0.01**			**0.002**
**FOLFOX vs HD-FUFA**	**0,380**	**0,184–0,787**	**0.009**	**0.266**	**0,119–0,595**	**0.001**
**FOLFIRI vs HD-FUFA**	**0,288**	**0,129–0,643**	**0.002**	**0.216**	**0,091–0,513**	**0.0005**
Target Therapy			0.370			0.84
Anti EGFR vs no biological agent	0.818	0.370–1.809	0.620	0.936	0,369–2,375	0.89
Anti VEGF vs no biological agent	1.049	0.482–2.281	0.905	1.165	0,507–2,678	0.72
**Levofolinate (Sodium vs Calcium)**	**0.564**	**0.401–0.795**	**0.001**	**0.5**	**0,347–0,720**	**0.0002**

In Cox multivariate analysis for PFS, NaLF halved the risk of progression - HR 0,5 - *p* value 0,0002: the only other significant factor in Cox multivariate was chemotherapy scheme: both FOLFIRI and FOLFOX decreased progression risk compared to HD-FUFA only therapy.

**Figure 5:**
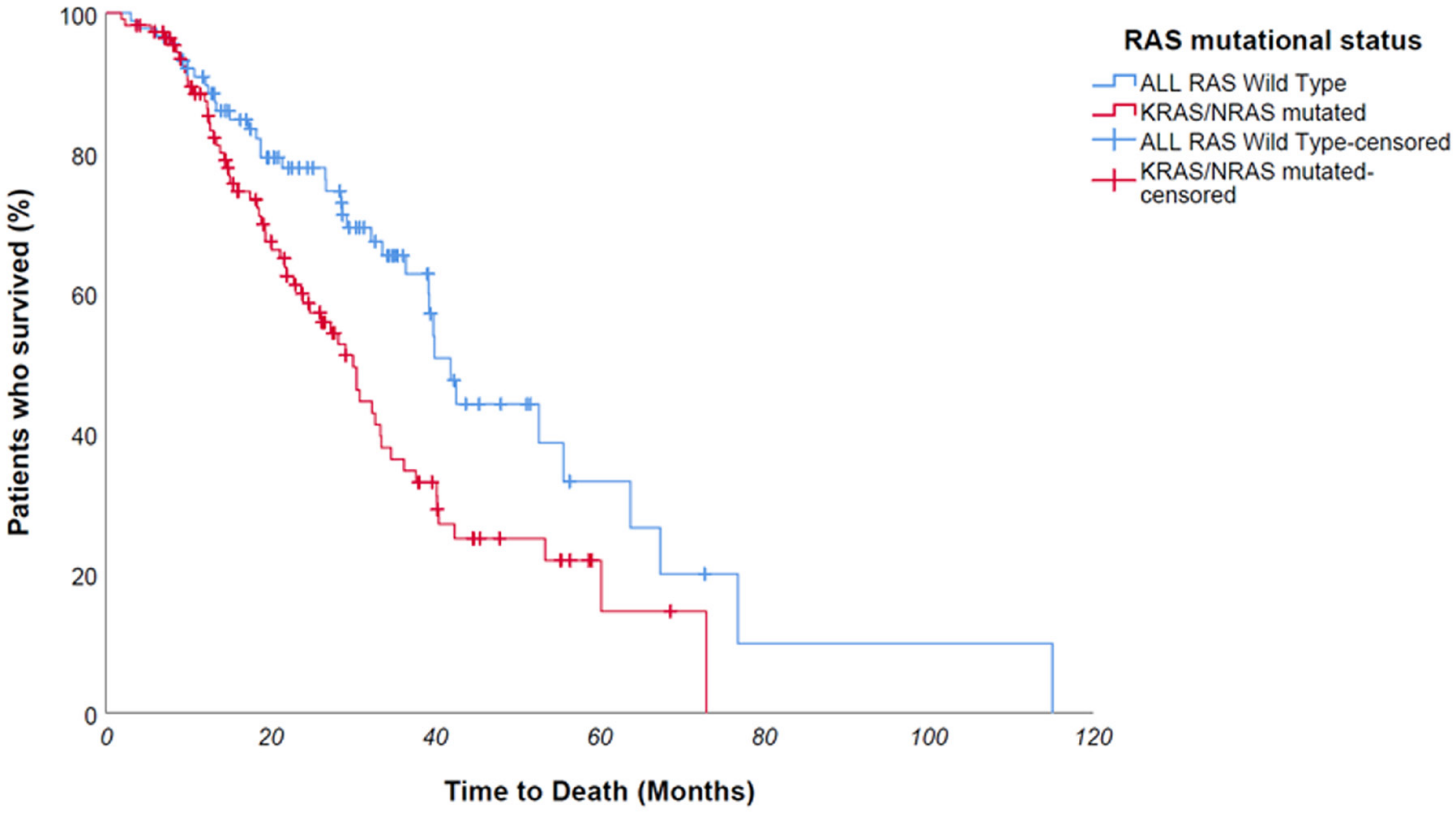
At Kaplan univariate analysis, OS was significantly longer for ALL RAS wild type patients compared to mutated counterpart - median 41,9 vs 30 months, *p* value 0,005.

**Figure 6:**
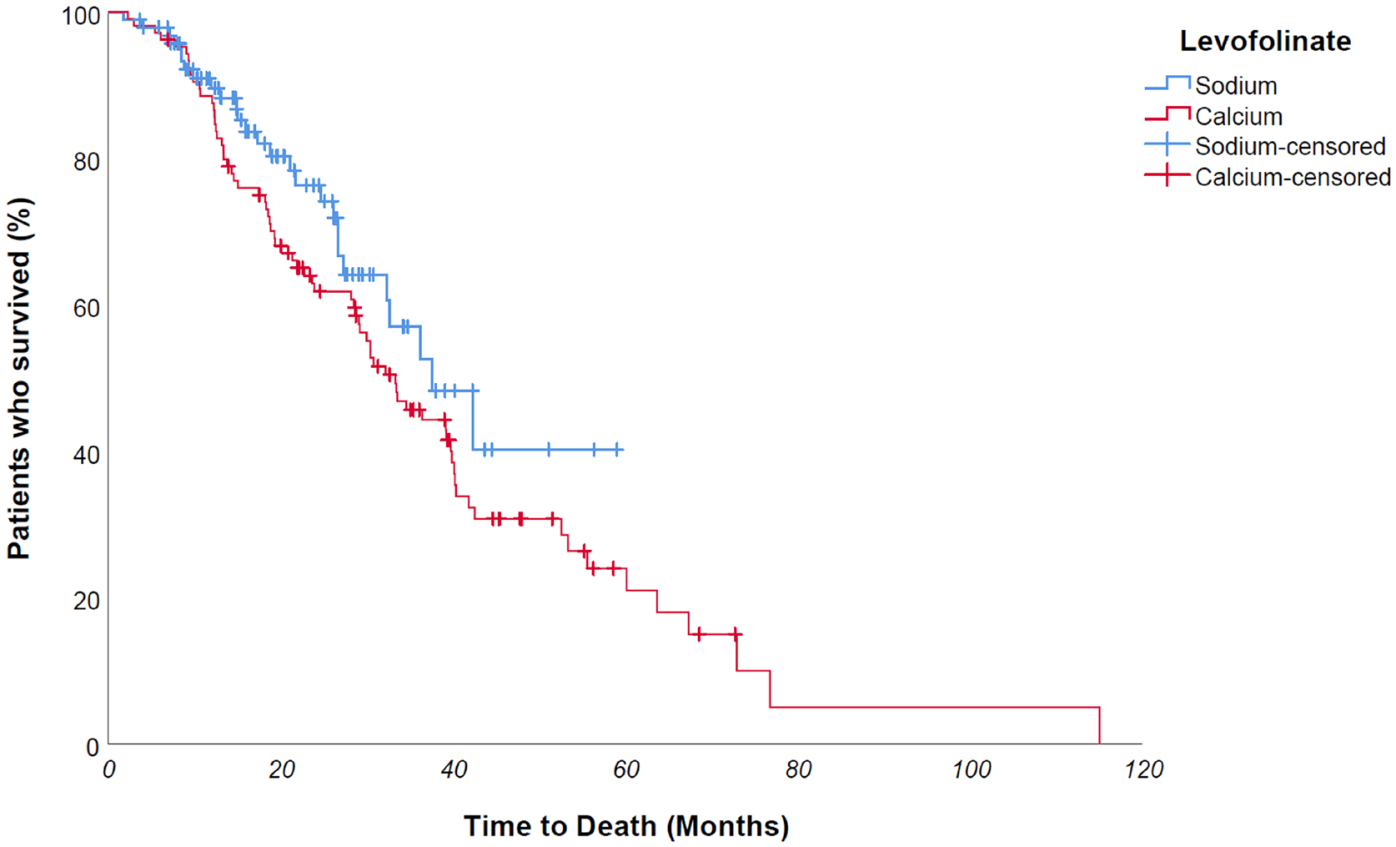
No difference was found in univariate OS analysis for folinic salt use - 37,7 vs 33,4 months - *p* value 0,151 - maybe due to therapy switch over of a large number of patients who underwent NaLF-based therapy in second line after first-line CaLF-based therapy, partially disguising the overall survival difference between the two groups.

At Kaplan univariate analysis, median overall survival of entire population was 34,6 months; OS was significantly longer for *KRAS/NRAS wild type* patients compared to mutated counterpart – median 41,9 vs 30 months, *p value 0,005* (Figure 5); furthermore, patients who had undergone metastases surgery achieved a longer survival – 115 vs 32,7 months – *p value 0,021*. No statistically significant difference was found in OS between Na-folinate and Ca-folinate groups – 37,7 vs 33,4 months – *p value 0,151* (Figure 6).

Factors affecting overall survival in Cox univariate analysis were *KRAS/NRAS* mutational status, metastates surgery and chemotherapy scheme: noteworthy, in multivariate analysis *KRAS/NRAS* mutational status and surgery lost their statistical significance, whereas chemotherapy scheme preserved it and levofolinate achieved it – HR 0,59 – *p value 0,03* (Table 3).

**Table 3:** OS Cox univariate and multivariate

Cox model	Univariate			Multivariate		
	HR	CI 95%	*p* value	HR	CI 95%	*p* value
Gender (M vs F)	1.13	0,745–1,715	0.565	1.101	0,713–1,700	0.665
Age at first line(continuous)	1.013	0.985–1.019	0.809	1.006	0,985–1,027	0.596
Tumor side (left colon vs right colon)	1.063	0.701–1.614	0.773	1,050	0,666–1,653	0.834
ALL RAS mutational status (WT vs mutated)	**0.56**	**0,370–0,847**	**0.006**	0.731	0,298–1,794	0.494
Metastases Surgery (Yes vs No)	**0.518**	**0.294–0.914**	**0.023**	0.58	0,321–1,048	0.071
**Chemotherapy scheme**			**0.003**			**0.004**
**FOLFOX vs HD-FUFA**	**0,327**	**0,131–0,817**	**0.017**	**0.350**	**0,127–0,963**	**0.042**
**FOLFIRI vs HD-FUFA**	**0,167**	**0,059–0,472**	**0.001**	**0.167**	**0,054–0,515**	**0.002**
Target Therapy			0.054			0.721
Anti EGFR vs no biological agent	0.716	0.297–1.722	0.455	0.905	0,305–2,682	0.857
Anti VEGF vs no biological agent	1.223	0.526–2.843	0.640	1.300	0,521–3,243	0.573
**Levofolinate (Sodium vs Calcium)**	0.718	0.456–1.130	0.152	**0.585**	**0,360–0,951**	**0.031**

Factors affecting overall survival in Cox univariate analysis were ALL RAS mutational status, metastates surgery and chemotherapy scheme: noteworthy, in multivariate analysis ALL RAS and surgery lost their statistical significance, whereas chemotherapy scheme preserved it and levofolinate achieved it - HR 0,59 - *p* value 0,03.

## DISCUSSION

The stock of therapeutic weapons available in mCRC has been progressively grown over the years, with improving both survival and patients’ clinical outcome: adding oxaliplatin to De Gramont regimen increased progression free survival from 6, 2 months to 9 months, although without a clear overall survival improvement [16]: capecitabine showed a comparable efficacy than infusional 5FU in combination with oxaliplatin [17]. With the introduction of so-called *molecular target drugs* into clinical practice, further improvements have been achieved in terms of survival: adding bevacizumab, the *first-in-class* antiangiogenic agent, to 5FU-FA based therapy, resulted in 10,6 median PFS versus the 6,2 months of *no bevacizumab* counterpart [18]. Afterwards, Saltz et al. demonstrated that the addition of bevacizumab to *oxaliplatin-based* chemotherapy regimens significantly improves PFS (11.1 vs 8.6 months), irrespective of RAS mutational status [19].

On the other hand, anti-EGFR agents improved PFS in *KRAS wild type tumor* affected patients: cetuximab in combination with FOLFIRI and panitumumab with FOLFOX led to an increase of PFS up to 9,9 and 10 months, respectively. FOLFIRI-cetuximab showed a PFS of 10.9 months in the KRAS/BRAF wild type subgroup, although without determining a statistically significant difference in PFS compared to the *FOLFIRI-only* treated group due to the low sample size [20, 21].

In our center experience, median PFS of patients was 12,8 months, slightly longer than previously reported: this discrepancy, although minimal, could be explained by the quite large percentage of patients who underwent surgery.

Increased survival for patients undergoing NaLF based therapy could be the consequence of a greater and more effective TS inhibition. A plausible reason for ALL RAS absence of significance on OS multivariate compared to univariate analysis can be found in extremely wide expression of TS in colon cancer cells. Indeed, on one hand we have an oncogene addiction toward EGFR pathway in ALL RAS wild type cells, but on the other hand we know that TS is essential for non-oncogenic pathways of cancer cells regardless of EGFR or RAS activation. Thus, we could postulate that the greater inhibition of TS we achieve with 5FU-LF combination, the lesser the role for the EGFR-driven oncogenic pathway. Noteworthy, there are coincidently more RAS mutated patients in NaLF group than the CaLF counterpart. Furthermore, as already shown, follow-up is shorter for NaLF-treated group, with fewer death events: probably, as events increase this analysis could become significant. Finally, we must highlight the therapy switch over of a large number of patients who underwent NaLF-based therapy in second line after first-line CaLF-based therapy, partially disguising the overall survival difference between the two groups.

## MATERIALS AND METHODS

This mono-institutional, observational study retrospectively reviewed medical records of 200 patients suffering from mCRC who underwent first-line therapy: patients did not provide any written informed consent, in accordance with the Declaration of Helsinki and Italian Privacy Protection Commissioner. All patients underwent 5-fluorouracil based chemotherapy: HD-FUFA (5FU bolus 400 mg/m^2^, calcium levofolinate 200 mg/m^2^, 5FU 48 hours-continuous infusion 2400 mg/m^2^) intravenous every 2 weeks, FOLFOX6 (5FU bolus 400 mg/m^2^, calcium levofolinate 200 mg/m^2^, 5FU 48 hours-continuous infusion 2400 mg/m^2^ and Oxaliplatin 85 mg/2) intravenous every 2 weeks, FOLFIRI (5FU bolus 400 mg/m^2^, calcium levofolinate 200 mg/m^2^, 5FU 48 hours-continuous infusion 2400 mg/m^2^ and Irinotecan 180 mg/2) intravenous every 2 weeks. When sodium levofolinate was used, it was co-administered along with 5FU continuous infusion, and 5FU bolus was administered just before elastomeric pump application; on the other hand, 5FU bolus was administered halfway through 2 hours calcium levofolinate infusion. Biological agents (antiangiogenic – bevacizumab or aflibercept, and anti-EGFR – panitumumab or cetuximab) were added to chemotherapy backbone depending on ALL RAS mutation status or clinical conditions by clinician choice.

Primary endpoints were Progression Free Survival (PFS) and Overall Survival (OS): PFS was defined as the interval between the first therapy administration and the date of disease progression or death for any cause; disease progression was defined as radiological tumor progression according to Response Evaluation Criteria in Solid Tumors, RECIST, version 1.1, or clinical progression, including death; OS was defined as the time from first-line therapy start to death from any cause.

Patients’ demographic and baseline characteristics, treatment patterns and acknowledged prognostic factors have been collected, with categorical variables being described by patient counts and percentages. Data were summarized as frequencies for categorical variables and mean and range values for continuous variables: Pearson chi-square test and Mann-Whitney Test for independent samples were used to compare such variables among groups, respectively.

Univariate analysis for median progression free- and overall survival was performed by Kaplan–Meier estimator: PFS and OS curves were obtained, and selected variables were compared using *two-sided log-rank test*. Hazard ratios (HR) were calculated by Cox Regression univariate and multivariate analysis: a *p* value ≤ 0.05 was considered statistically significant. The SPSS statistical package version 26.0 (SPSS Inc., Chicago, IL) was used for all statistical analysis.

## CONCLUSIONS

Finally, in our experience concomitant administration of 5FU and sodium levofolinate prolongs the time to disease progression of patients suffering from mCRC, moreover decreasing death risk by 40%. Further prospective, head-to-head trials are warranted in order to confirm these findings, although in our retrospective experience the use of sodium levofolinate showed a clear clinical benefit for patients, regardless of RAS mutational status, tumor side or biological agent added to chemotherapy.
